# The Future Abundance of Key Bird Species for Pathogen Transmission in the Netherlands

**DOI:** 10.1007/s10393-025-01727-9

**Published:** 2025-07-04

**Authors:** Martha Dellar, Henk Sierdsema, Maarten Schrama, Gertjan Geerling, Peter M. van Bodegom

**Affiliations:** 1 Deltares, Daltonlaan 600, 3584BK Utrecht, The Netherlands; 2 Institute of Environmental Sciences, University of Leiden, Van Steenis Building, Einsteinweg 2, 2333CC Leiden, The Netherlands; 3 Sovon Dutch Centre for Field Ornithology, Toernooiveld 1, 6525 ED Nijmegen, The Netherlands; 4 Department of Environmental Science, Radboud University, Nijmegen, The Netherlands

**Keywords:** Netherlands, pathogens, birds, random forest, scenarios, disease risk

## Abstract

Wild birds serve as reservoirs and vectors for many different pathogens. Changes in their distribution and abundance, due to environmental change, will influence disease risk. We investigated potential changes in abundance for three commonly occurring species that are likely major drivers of a wide range of diseases: blackbirds, mallards and house sparrows. These are competent hosts for avian influenza and West Nile virus, among other pathogens. Using the Netherlands as a case study, we created random forest models for predicting the distribution and (relative) abundance of these species, both now (1991–2020) and in the future (2036–2065). The three species had different spatial distributions, largely related to their preferred habitat and food availability. In the future, mallard and house sparrow populations were predicted to increase, while there was little change for blackbirds. These changes in abundance have a potentially strong relationship with disease risk, since species abundance is linked to the size of pathogen reservoirs. We demonstrate this relationship by linking blackbird abundance to cases of Usutu virus in the Netherlands. Our work illustrates the potential value of forecasting (relative) abundance to estimate future disease risk and to assist planning of disease management actions.

## Introduction

Wild birds can serve as both reservoirs and mechanical vectors for many different pathogens, with health implications for humans, domestic animals, other wild animals and the birds themselves (Reed et al., 2003; Tsiodras et al., 2008; Levison, 2016). In Northwestern Europe, recent outbreaks of Usutu virus (USUV), West Nile virus (WNV) and avian influenza (AI) have highlighted the risk of bird-borne pathogens and the ease with which they can spread (Oude Munnink et al., 2020; Sikkema et al., 2020; Vlaskamp et al., 2020; Caliendo et al., 2024). Changes in both climate and land use lead to changes in the distribution and abundance of birds (Jetz, Wilcove and Dobson, 2007; Kampichler et al., 2012; Howard et al., 2015), implying that future risks of bird-borne diseases may strongly deviate from current spatial patterns in risk. However, previous studies of European bird distribution have not focused on key bird species in relation to diseases and have generally not considered knock-on effects on pathogen transmission (Jetz, Wilcove and Dobson, 2007; Kampichler et al., 2012; Howard et al., 2015; Hendriks et al., 2016). Given the lack of knowledge on the future distribution of key bird species for pathogen transmission, it is highly valuable to have future predictions for the distribution and abundance of such species. Such predictions can be used in modelling studies to estimate future disease risk (De Wit et al., 2024a, b) and provide tools for acting upon such predicted changes for risk control.

We know several species which are likely to make a significant contribution to the spread of certain diseases in Northwestern Europe; these are common blackbirds (*Turdus merula*), house sparrows (*Passer domesticus*) and mallards (*Anas platyrhynchos*) (Ashraf et al., 2015; Beer et al., 2021). By focusing on a particular region and a few key species, we can gain a better understanding of the role of shifting bird distributions on pathogen transmission. These species all have large breeding populations in Northwestern Europe, are collectively capable of transmitting a wide range of pathogens and were not included in recent efforts to make predictive abundance maps for a suite of different bird species across Europe (Hendriks et al., 2016). They are also all commonly found in urban and peri-urban areas, meaning that they will often be in close contact with people, with possible consequences for human health. Blackbirds are part of the *Turdidae* family. They are highly susceptible to USUV, which has caused significant reductions in the European population in recent years (van den Bremer and van Turnhout, 2021; Siljic et al., 2023). Blackbirds are also susceptible to avian malaria (Agliani et al., 2023), competent hosts of WNV (Angelou, Kioutsioukis and Stilianakis, 2021) and a key reservoir species for Lyme borreliosis in central Europe (Taragel’ová et al., 2008). House sparrows are part of the *Passeridae* family. They are competent hosts for WNV, AI and avian malaria (Medeiros et al., 2016; Shriner and Root, 2020), as well as bacterial diseases such as *Escherichia coli* and *Salmonella spp.* (Vilela et al., 2012). Mallards are part of the *Anatidae* family. They are competent hosts for WNV and AI and have the potential to spread pathogens over long distances as part of their migratory movements (Kilpatrick, LaDeau and Marra, 2007; Pérez-Ramírez, Llorente and Jiménez-Clavero, 2014; van Toor et al., 2018).

In this study, we aim to determine how climate change and land-use change affect the distribution and abundance of these three key bird species. We will also consider the effects these changes might have on pathogen transmission. In particular, we will analyse the case of USUV in the Netherlands and demonstrate the link between blackbird abundance and disease incidence. We use the Netherlands as a case study, since there are both large amounts of bird data available and extensive environmental data. In addition, new detailed socio-economic scenarios have recently been published for this country (Dellar et al., 2024a; Dellar et al., 2024b), providing detailed information on potential future land-use changes. For all three species under consideration, it is estimated that there are at least 180,000 breeding pairs in the Netherlands, and for house sparrows there may be up to a million (Sovon, 2023). Also, the Netherlands has recently experienced a significant USUV outbreak as well as its first introduction of WNV (Sikkema et al., 2020; van den Bremer and van Turnhout, 2021).

By identifying trends in the abundance and distribution of these species, we have the opportunity to identify areas which may benefit from additional surveillance in future, thanks to changing population patterns. The predictions can also be used to model avian-borne pathogens and identify areas of high risk. Such areas can then be targeted as part of disease-prevention efforts, for example by warning health services to be particularly alert for certain symptoms.

## Methods

### Overview

We trained a random forest model and used this to make predictions for the relative abundance of blackbirds, mallards and house sparrows in the Netherlands, for both the current situation (1991–2020) and the future (2036–2050), based on different scenarios. For our response variable, we used bird count data from both the Netherlands and France. The current French climate is similar to the future Dutch climate (Bastin et al., 2019), so by including the French data we can minimise extrapolation for future predictions beyond our training data. We used a variety of explanatory variables, representing weather, land use, vegetative cover and soil properties.

### Bird Count Data

Bird data for the three species under consideration for the Netherlands were taken from the Meetnet Urbane Soorten (MUS), Meetnet Agrarische Soorten (MAS) and Broedvogel Monitoring Project (Common Bird Census, CBC) datasets (Teunissen et al., 2019; Schoppers et al., 2020; Vergeer et al., 2023). For MUS and MAS, point count data are collected during three or four visits in the breeding season by volunteers in urban and rural areas, respectively. They count all the breeding birds they see during a five (MUS)- or ten (MAS)-minute period. For MUS, measurements are taken between one hour before and two hours after sunrise, while for MAS they are taken up to five hours after sunrise. Some measurements were taken outside the breeding season and these were excluded. For CBC, volunteers walk a set route five to ten times within a prescribed area, recording all the birds, or a preset selection of species, they see or hear. This only happens during the breeding season and generally during the first five hours after sunrise. The data are then converted to a 250 m grid. The monitoring areas cover all major land-use types in the Netherlands. The breeding seasons of the three species used are shown in Table 1. We excluded measurements taken in the same location in the same year within each dataset, keeping just the maximum value, since this minimises underestimation due to failures of detection.

**Table 1:** Breeding season per species (van Turnhout, 2011).

Species	Breeding season
Blackbird	March 1–July 15
Mallard	April 1–June 10
House sparrow	April 10–June 19

Bird data for France were taken from the Suivi Temporel des Oiseaux Communs (STOC) dataset (Vigie-Nature, 2021). Point count data are collected by volunteers during the breeding season, counting all the birds they see or hear during a five-minute period. Measurements are taken one to four hours after sunrise. Again, we excluded measurements taken in the same location in the same year.

### Filtering and Selection of Bird Count Data

**Figure 1:**
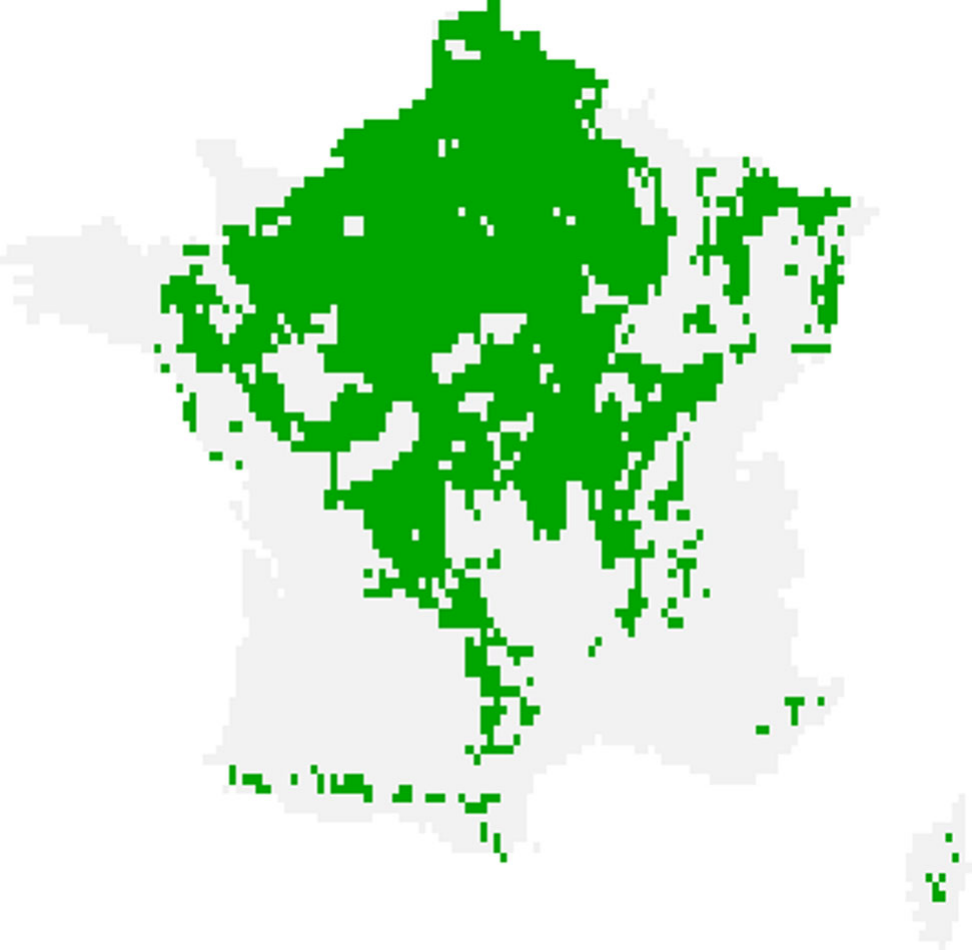
French area from which bird data were taken for our study (green area). The current climate and elevation in this area was deemed sufficiently similar to the Dutch climate and elevation in 2050.

We used data from France since the current French climate is comparable to the Dutch climate in 2050 (Bastin et al., 2019) and we wanted to avoid extrapolation when making future predictions for the Netherlands. We only took French bird data from areas where the climate was sufficiently similar to the expected future Dutch climate. To estimate the future Dutch climate, we used summarised statistics from future climate scenarios produced by the Dutch Meteorological Association (KNMI, 2023). These are included in the supplementary materials (‘Climatic ranges’) and include average minimum, mean and maximum temperatures as well as total rainfall for different seasons, with a total of ten variables. For each climatic variable, a range was given for 2050. We selected areas of France which fell in or near these ranges, while still capturing an area which covered the more extreme values which are expected due to increased future climatic variability. To this end, we chose the areas of France where, for at least five of the ten variables, the average climate was within the given range, plus or minus an additional margin equal to the range’s length, to account for an increase in extreme values. This meant that we excluded those areas where the climate was dramatically different to the Netherlands. French climatic data were taken from the E-OBS dataset (Cornes et al., 2018), using ensemble mean data on a 0.1° grid. The French area deemed to have a comparably similar climate to the Netherlands in 2050 is shown in Fig. 1. Finally, we removed any remaining French datapoints with an altitude above 350 m, since this is the highest point in the Netherlands and we are only interested in French areas which are comparable.

**Figure 2:**
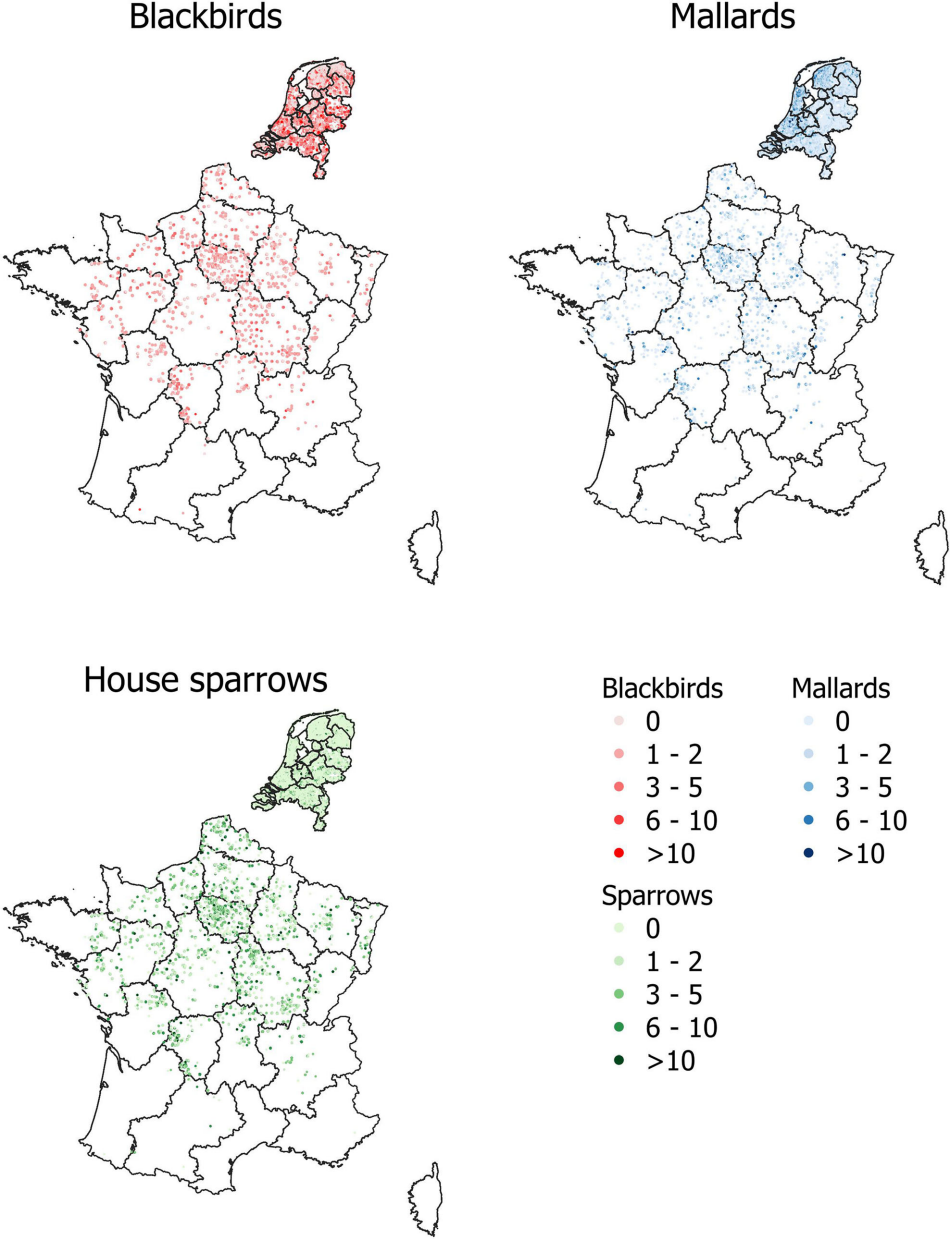
Point locations of observational data used to train our model. Darker shades indicate that more birds were observed. Dutch data were collected between 1983 and 2023, while French data were collected between 2001 and 2023.

Both the Netherlands and France have experienced recent Usutu outbreaks which have had a particularly large effect on the blackbird population (Lecollinet et al., 2016; Oude Munnink et al., 2020). To avoid this affecting our results, we discarded blackbird data from after 2015 for France, and from after 2016 for the Netherlands. The final number of datapoints per dataset is shown in Table 2, and their locations are shown in Fig. 2.

**Table 2:** Number of datapoints per dataset.

Dataset	Country	Number of presences	Number of absences	Time period
Meetnet Urbane Soorten (MUS)	Netherlands	Blackbirds: 50,794 Mallards: 14,186 House sparrows: 24,758	Blackbirds: 9123 Mallards: 44,476 House sparrows: 34,386	2007–2023
Meetnet Agrarische Soorten (MAS)	Netherlands	Blackbirds: 1228 Mallards: 2630 House sparrows: 626	Blackbirds: 3215 Mallards: 1807 House sparrows: 3813	2000–2023
Common Bird Census (CBC)	Netherlands	Blackbirds: 32,742 Mallards: 119,431 House sparrows: 10,798	Blackbirds: 40,382 Mallards: 133,561 House sparrows: 194,351	1983–2023
Suivi Temporel des Oiseaux Communs (STOC)	France	Blackbirds: 33,986 Mallards: 2072 House sparrows: 11,110	Blackbirds: 11,653 Mallards: 3522 House sparrows: 1191	2001–2023

These are divided into presences and absences for convenience, though in our analysis we used count data.

### Environmental Predictor Data

We used a wide range of environmental predictors for estimating the distribution and abundances of the key bird species, based on the literature (Kampichler et al., 2012; Howard et al., 2015; Hendriks et al., 2016) and expert advice from the Dutch Centre for Field Ornithology (Sovon). Weather data were taken from the E-OBS dataset (Cornes et al., 2018), using ensemble mean data on a 0.1° grid. Climatic parameters were calculated over two time periods: The breeding season for the year the measurement was taken (see Table 1) and the winter preceding the breeding season (December 1^st^–February 28th). Parameters used were mean, minimum and maximum temperature, temperature range, number of days with a minimum temperature below 0 °C, total precipitation, precipitation coefficient of variation and total precipitation during the driest four-week period. While many other studies have used very general climate data (Kampichler et al., 2012; Howard et al., 2015; Hendriks et al., 2016), often averaged over thirty-year periods, it has been found that using shorter time scales (i.e. weather), as we do here, produces better results when modelling bird distributions. Reside et al., (2010) found that climate-based models tend to overestimate suitable habitat when compared with weather-based models.

Land-use data were taken from the CORINE dataset (CLMS, 2020a). This is available on a 100 m grid for the years 1990, 2000, 2006, 2012 and 2018. For each bird observation, we used land-use data from the closest CORINE year. CORINE uses 44 different land-use classes. Many of these are quite similar, and it is efficient to group them together. The grouping we used is provided in the supplementary materials (‘Deriving future land use’, Table S1). Since birds can travel a reasonable distance, different sized buffers around each observation were considered. The proportions of each land use type within a radius of 500 m, 1 km and 2 km of each observation were used as covariates in our model. We also used tree density and the proportion of grass cover within these same radii. These were taken from the Copernicus Land Monitoring Service and were available on a 100 m grid (CLMS, 2020b, c).

We also used the proportions of clay and sand in the soil, from the dataset of topsoil physical properties for Europe on a 500 m grid (Ballabio, Panagos and Monatanarella, 2016) provided by the European Soil Data Centre (ESDAC, 2025; Panagos et al., 2012). Altitude was also included and was taken from the European Environment Agency’s elevation map on a 1 km grid (EEA, 2016). Finally, we included the dataset from which the training data were taken, to account for variations between our bird data sources.

### Model Fitting and Evaluation

We modelled the relative abundance of each species by fitting the count data (including observed absences) to the environmental data described above using a random forest. We chose to use a random forest model since it captures nonlinear relationships, deals well with a large number of covariates without over-fitting and does not make assumptions about the underlying data distribution. Other studies have found it to be a good choice for modelling bird abundance (Palacio, 2018; Kosicki, 2020). We used 500 trees and a minimum node size of 5. Inverse distance weighting was used to interpolate the response residuals. We used the SDMaps package (Kampichler, Hallman and Sierdsema, 2020) in R (R Core Team, 2021) to implement the random forest.

We evaluated our models by calculating the mean average error (average magnitude of difference between predictions and observations), mean forecast error (average signed difference between predictions and observations), root mean-squared error (square root of the average squared difference between predictions and observations), Pearson correlation and explained variance. In addition, observed values were regressed on predicted values, and the intercept and slope were calculated, to test for bias in our predictions. We also calculated the variable importance (as a percentage) for all environmental predictors, based on the mean decrease in impurity. We calculated the effects of uncertainty in the model fitting process using a bootstrapping procedure with 200 iterations (trial and error showed this was sufficient to achieve convergence). We made predictions for the 1991–2020 period for each iteration and then calculated the 95% confidence interval per grid square across the iterations.

### Scenarios

For each species, we made predictions of relative abundance during the breeding season on a 1 km grid, averaged over the time periods 1991–2020 and 2036–2065. This allowed us to see large-scale trends in how the populations are changing thanks to climate and land-use change. This is useful for disease outbreak preparedness in that it can highlight areas which may not previously have been considered for surveillance but which may become relevant in the future. It is also helpful as input for models of future disease risk, allowing the identification of areas which may be particularly at risk and enabling targeted interventions. We make average predictions over these time periods rather than year-on-year predictions, due to uncertainties about specific climatic and land-use changes and their timing. It is not possible to calculate absolute abundance from the available data, so the results for each species were scaled to have a maximum of 1.

Altitude and soil parameters remained unchanged when making future predictions. For the 1991–2020 period, we used the CORINE land-use map for 2006 (the closest year to the centre of the period) and grass cover and tree density maps from 2018 (CLMS, 2020a, b, c). For the 2036–2065 period, land-use parameters, grass cover and tree density were derived from the Dutch One Health SSPs (Shared Socio-economic Pathways) (Dellar et al., 2024a; Dellar et al., 2024b). See the supplementary materials on Dryad (‘Deriving future land use’) for full details of how this derivation was performed. The Dutch One Health SSPs are socio-economic scenarios recently developed for the Netherlands with a focus on health. They are based on the global SSP scenarios (O’Neill et al., 2017) and include SSPs 1, 3, 4 and 5. They include land-use maps for 2050. Future climate data were taken from scenarios published by the Dutch Meteorological Organisation (KNMI, 2023). They provide six scenarios: low, medium and high emissions, each with a wet and dry variant. The different emissions levels are approximately equivalent to RCPs (Representative Concentration Pathways) 2.6, 4.5 and 8.5, respectively. Each scenario is the result of an 8-model ensemble and uses 1991–2020 as a reference period. The scenarios are on a 12 km grid, which we converted to a 1 km grid using bilinear interpolation. We paired the low emissions scenario with SSP1, medium emissions with SSP4 and high emissions with SSPs 3 and 5. We averaged over the eight ensembles and also averaged the wet and dry variants. For each weather variable, we calculated the values for each grid cell and each year in the 30 year period, then averaged over the years.

We tested for statistically significant (*p* < 0.05) differences between our predictions for different species and scenarios using an ANOVA. We confirmed the assumptions of normality and homogeneity of variance of the residuals and then performed the Bonferroni multiple-comparison test. We also calculated the Pearson correlation between the scenario predictions within each species, to check for spatial similarity.

### Case Study: USUV in Blackbirds

While it seems intuitively likely that the abundance of a competent host for a disease is linked to the risk of an outbreak of that disease, we are not aware of any study testing this connection. To this end, we examined the case of blackbirds and USUV in the Netherlands. USUV is a mosquito-borne virus and was first detected in the Netherlands in 2016, with the outbreak starting in the south and spreading northwards over a period of several years (Rijks et al., 2016; de Wit et al., 2024a, b). This led to the nationwide blackbird population being reduced by nearly a third (van den Bremer and van Turnhout, 2021). We used data on USUV cases from both live and dead blackbirds, collected via citizen science. Live birds were captured by trained volunteer ornithologists, and throat and cloacal swabs were taken, as well as blood and feather samples. Samples were also taken from dead birds reported by the general public. Full details of the sampling and testing process can be found in Münger et al. (2024). This provided us with 106 positive cases of USUV in live blackbirds and 208 cases in dead blackbirds, as well as 4500 negative cases (4309 live, 191 dead), sampled between 2016 and 2022. We plotted the positive cases and visually compared them with our predicted blackbird distribution for 1991–2020. We also compared the bird abundance values in grid cells where positive USUV cases were found with bird abundance for the whole country, using the Wilcoxon rank-sum test. This enabled us to check if blackbird abundances were high in places where USUV had been found. We also checked the converse, i.e. is USUV found in areas with a high abundance of blackbirds? For this we used logistic regression with tenfold cross-validation. We converted the USUV case data to a 1 km grid representing presence/absence and used this as our response variable. We restricted the USUV case data to cases found between July and October, since 88% of positive cases came from this period and it coincides with the mosquito season. This left us with 198 ‘presence’ gridcells and 144 ‘absence’ gridcells. The explanatory variables (also on 1 km grids) are shown in Table 3 and represent the key factors necessary for transmission of a vector-borne disease (i.e. a host, a vector and the presence of the disease itself) as well as temperature, which affects mosquito population dynamics, extrinsic incubation period and biting rate (de Wit et al., 2024a, b).

**Table 3:** Explanatory variables used in the logistic regression analysis for the Usutu virus (USUV) case study.

Variable	Justification	Source
Predicted blackbird distribution for 1991–2020	Common host species of USUV	This study
Peak seasonal *Culex pipiens* abundance for 1991–2020	This is the mosquito species largely responsible for USUV transmission in the Netherlands	Krol et al. (2024)
Binary variable showing if USUV had previously been found within 20 km in the same year (in any species)	Since outbreaks are only possible when the virus is present	Münger et al. (2024)
Mean temperature in the warmest quarter	Highly relevant for disease transmission and mostly overlaps with the months under consideration	BioClim: Fick and Hijmans, (2017)

## Results

### Model Performance

The model performance metrics of the random forest models for each of the three species are shown in Table 4. All models performed well in predicting relative abundance, with the blackbird model having a particularly high correlation between predicted and observed values. Errors were generally low, and the fact that all MFEs were close to zero suggests that there was little bias in the predictions. On the other hand, the negative intercepts and slopes greater than 1 suggest that the models may have a tendency to underpredict lower values and overpredict higher values. The blackbird model had the highest explained variance, suggesting that the chosen predictors were particularly effective at representing the ecological processes influencing this species. The lower explained variance of the other species, particularly mallards, suggests that different or additional predictors may have been preferable to capture their associations with their environment.

**Table 4:** Model performance metrics (MAE, mean average error; MFE, mean forecast error; RMSE, root mean-squared error).

Performance metric	Blackbirds	Mallards	House sparrows
MAE	0.325	0.236	0.340
MFE	− 0.012	− 0.015	− 0.020
RMSE	0.544	0.603	0.896
Pearson correlation	0.954	0.791	0.726
Intercept	− 0.231	− 0.158	− 0.170
Slope	1.127	1.204	1.146
Explained variance	69.7	53.8	60.2

Intercept and slope are based on a regression of observed values on predicted values.

### Key Variables Affecting Bird Abundance

Given the large number of predictors used, we grouped them by type (climate, land use or vegetation) and summed the variable importances within each group. This enables us to understand the contribution of each category, rather than focussing on individual predictors. Each category tells us something about what is important to a given bird species. For example, land-use predictors highlight the role of habitat availability and human influence (among others). Climatic predictors might relate to food availability, metabolic costs and habitat suitability, while vegetative predictors might relate to the availability of nesting sites, shelter and food. Details of the contributions of each individual predictor, including partial dependence plots, are available in the supplementary materials. It should be noted that, while this grouping approach provides insights into broad patterns, categories with a large number of predictors may have an artificially inflated importance. The results are shown in Table 5.

**Table 5:** Grouped variable importance (as a percentage) for each bird species model.

		Blackbirds		Mallards		House sparrows	
Climate	Temperature (10)	20.36	12.44	35.09	21.43	24.49	15.13
	Precipitation (6)		7.92		13.65		9.36
Land use	Urban (12)	37.05	14.19	36.35	11.68	36.86	11.84
	Agriculture (12)		10.91		13.83		14.71
	Nature (9)		6.93		5.35		6.08
	Water and wetlands (9)		5.02		5.49		4.24
Vegetative cover	Grass cover (3)	22.37	8.64	15.97	7.43	18.75	8.01
	Tree cover (3)		13.73		8.55		10.74
Other	Soil content (2)	20.22	7.84	12.59	6.59	19.90	5.66
	Altitude (1)		4.03		4.45		5.04
	Dataset ID (4)		8.36		1.55		9.20

The numbers in brackets indicate the number of variables in each group.

### Current Abundance

**Figure 3:**
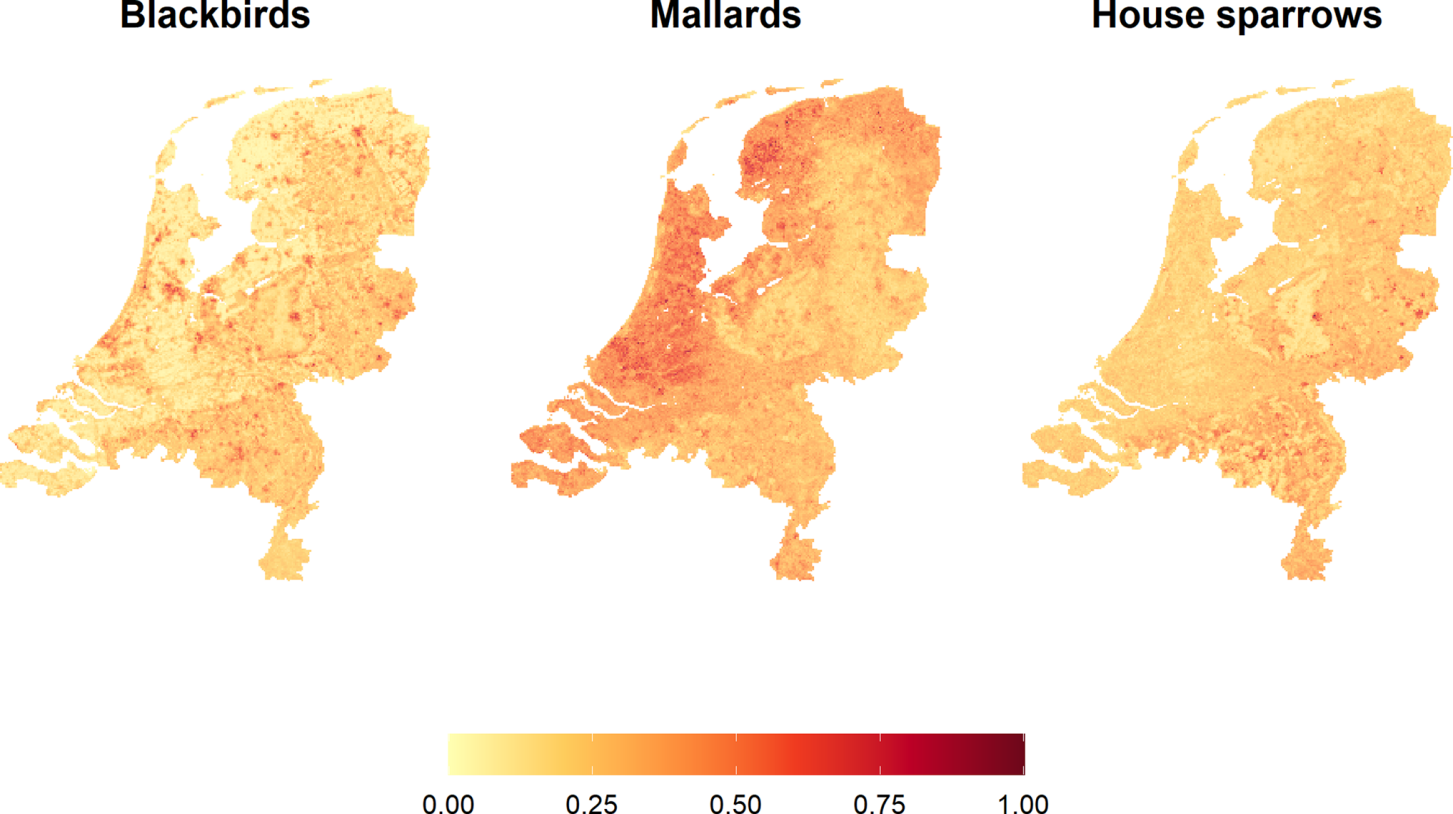
Relative breeding bird abundance in the Netherlands during the breeding season for the period 1991–2020.

The relative breeding season abundance for the three species for the period 1991–2020 is shown in Fig. 3. Blackbirds were generally found to be more abundant in the eastern part of the country though the highest abundances were in urban areas and along the western coast. Mallards on the other hand showed higher abundances in the west. Sparrows were more evenly distributed across the country, though abundances were slightly higher in the south-east, particularly in south-eastern cities, and were noticeably lower in large natural areas.

### Future Abundance

**Figure 4:**
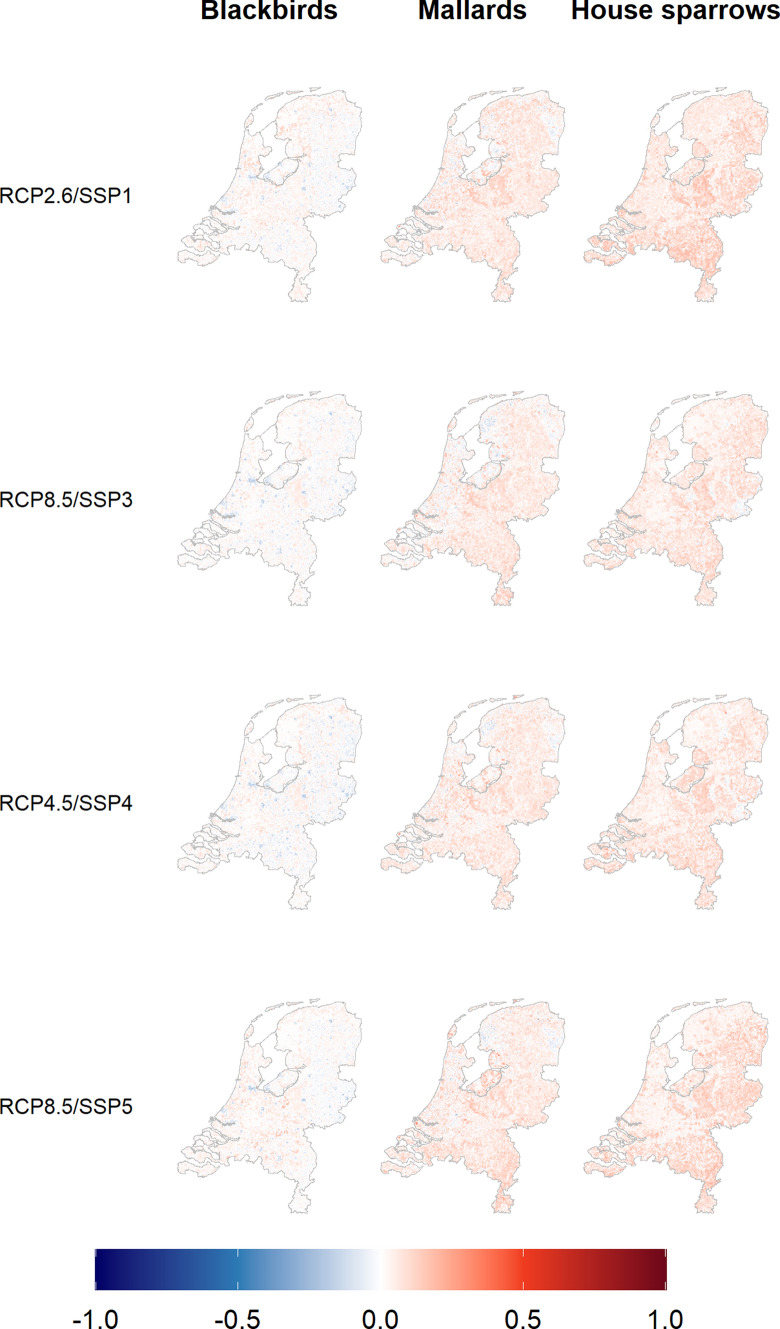
Change in the relative abundance of three bird species in the Netherlands during the breeding season from present (1991–2020) to future (2036–2065) for each of 4 scenarios. Future distributions were scaled by the same factors as current distributions (Fig. 3), and the difference between these and current distributions was calculated. RCP (Representative Concentration Pathway) indicates the climate scenario used, and SCP (Shared Socio-economic Pathway) indicates the socio-economic scenario used. White indicates no change from the current situation, while blue indicates a decrease in abundance, and red indicates an increase. The changes shown here are summarised in Table 5.

The change in relative abundance between the periods 1991–2020 and 2036–2065 is shown in Fig. 4 and summarised in Table 6. In general, blackbird numbers are expected to stay roughly the same, while the abundances of mallards and house sparrows are expected to increase. Increases are generally larger (relative to current values) in places where abundance is currently low. Future abundance was always significantly higher (*p* < 0.0001) than in the reference period, except for blackbirds for SSPs 3 and 4, where there was no significant difference (*p* > 0.9). The results for the different scenarios show similar spatial patterns within each species, though with some variation in the magnitude of the changes. The correlation between the scenarios was always above 0.90, 0.82 and 0.77 for blackbirds, mallards and house sparrows, respectively. Within each species, the differences between scenarios always had *p* < 0.0001, with the following exceptions: for blackbirds SSP1 vs 5 and SSP3 vs 4 were not significant (*p* = 0.18), for mallards SSP 1 vs 4 and SSP1 vs 5 had significant differences with *p* < 0.05, and for sparrows SSP3 vs 4 were significantly different with *p* < 0.001. Within scenarios, the difference between species was always significant with *p* < 0.0001.

**Table 6:** Summary of changes in relative abundance from present (1991–2020) to future (2036–2065).

		1st quartile	Mean	3rd quartile
RCP2.6/SSP1	*Blackbirds* *Mallards* *House sparrows*	− 0.02 0.02 0.04	0.01 0.06 0.09	0.03 0.10 0.13
RCP8.5/SSP3	*Blackbirds* *Mallards* *House sparrows*	− 0.02 0.01 0.03	0.00 0.05 0.06	0.02 0.09 0.10
RCP4.5/SSP4	*Blackbirds* *Mallards* *House sparrows*	− 0.02 0.02 0.02	0.00 0.05 0.07	0.03 0.09 0.11
RCP8.5/SSP5	*Blackbirds* *Mallards* *House sparrows*	− 0.01 0.02 0.03	0.01 0.06 0.08	0.03 0.10 0.12

A value of 0 indicates no change. Other values are proportions of the maximum abundance across the country for the present period.

### Model Uncertainty

Uncertainty due to the model generation process was calculated. Using the same scaling as for the current abundance maps (Fig. 3), the average confidence intervals for the whole country were ± 0.04 for blackbirds ([mean—0.04, mean + 0.04] where the mean is different for each grid cell), ± 0.08 for mallards and ± 0.06 for house sparrows. Detailed results are available in the supplementary materials.

### Case Study: USUV in Blackbirds

**Figure 5:**
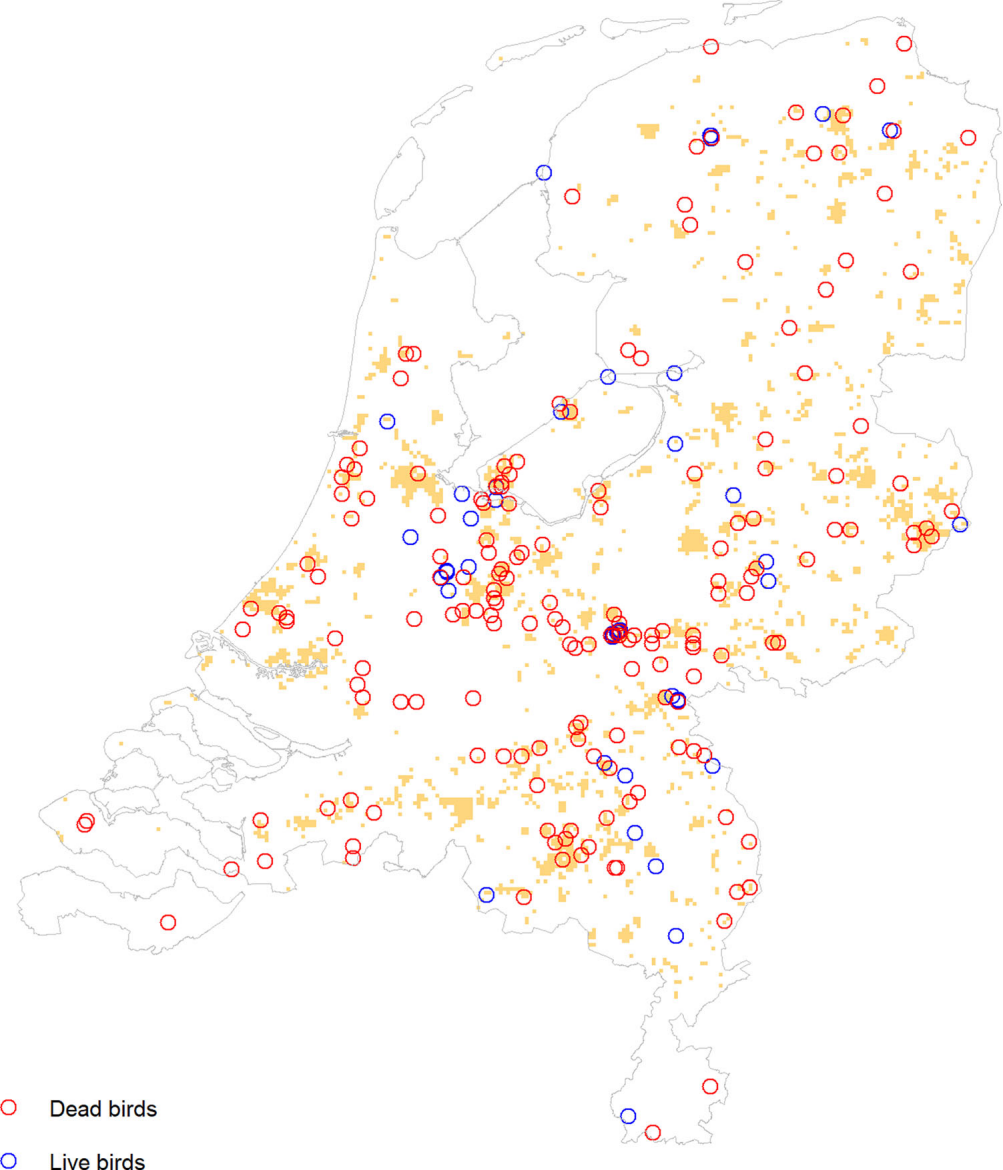
Point locations of positive Usutu virus cases found in blackbirds in the Netherlands between 2016 and 2022. The yellow shading indicates areas where the current blackbird abundance is predicted to be above 0.35 (from Fig. 3).

Figure 5 shows the locations of the positive cases of USUV found in blackbirds from 2016 to 2022. The yellow areas are locations where the current blackbird abundance is predicted to be high (from Fig. 3). It can be seen that the positive USUV cases were typically found in places of high blackbird abundance. Moreover, grid cells with at least one positive USUV case had significantly higher predicted blackbird abundance than the country as a whole (*p* < 0.001, mean abundance for USUV-positive grid cells was 0.30, compared with 0.20 for the national average). When fitting the logistic regression, it was found that the model had an accuracy of 61.6% (kappa = 0.18), indicating that the model worked better than chance. The most important explanatory variable was the presence of USUV within 20 km (relative importance = 1), followed by temperature (0.66) and blackbird abundance (0.55); mosquito abundance was unimportant. There was a trend suggesting that blackbird abundance had a positive effect on USUV cases (*p* = 0.053). While this model does not have high predictive power, it nevertheless demonstrates that blackbird abundance can be a useful predictor of USUV cases. Full results are available in the supplementary materials.

## Discussion

We created random forest models to predict the relative abundance of three bird species (blackbirds, mallards and house sparrows) which are likely to be especially relevant for disease transmission in the Netherlands, given their large populations and susceptibility and competence for a wide range of diseases (Ashraf et al., 2015; Beer et al., 2021; Sovon, 2023). We used these prediction models to project the abundance of these species under different future scenarios, which included climate, land-use and vegetation factors. We also considered the case of USUV in Dutch blackbirds, demonstrating both the link between abundance and disease incidence, and the utility of our predictions as a tool for identifying areas of high risk.

Our results show that, besides climate, land use and vegetative cover (and predicted changes therein) are particularly important predictors of bird abundance, with the latter two being more important than climate. Other studies at a European scale have found similar results (Kampichler et al., 2012; Howard et al., 2015; Hendriks et al., 2016), though have often found that climate is the most important predictor. This might be explained by the fact that the Netherlands is a small country and experiences relatively little spatial climatic variation, while at the same time, the high population density (compared with most other countries) leads to a higher prevalence of anthropogenic land-use types. It is also possible that some of our variables were correlated with one another, which could potentially affect the relative importance of different variables, though we deem this unlikely thanks to our use of a random forest model and grouped categories to examine variable importance. Temperature was more important than precipitation in predicting bird abundances, possibly because it has many direct and indirect effects on birds, influencing food availability, breeding success, migration, habitat, predators and competition, among others (Howard et al., 2015). Similarly, land use and vegetative cover have significant effects on both food availability and nesting opportunities (Kampichler et al., 2012). This highlights the need to include a wide range of factors when investigating bird abundance and disease risk.

Looking at current abundance, there is a different pattern for each species. Blackbirds and sparrows have higher abundances in the eastern parts of the country, where there is high soil sand content and greater tree cover, while mallards have higher abundances in the west, where there is high soil clay content and more urban areas. Blackbirds also have very high abundance in urban areas throughout the country, while house sparrows seem to prefer the eastern cities and to particularly avoid large natural areas. These results are in line with previous research. Blackbirds have been becoming increasingly urbanised for several decades in the Netherlands, largely as an alternative to migration. This is thought to be due to a combination of climate change and urban areas offering a warmer microclimate and better winter food availability (von dem Bussche et al., 2008; Van Vliet, Musters and Ter Keurs, 2009; Evans et al., 2010; Møller et al., 2014). Blackbirds have also been found to prefer forests and woodlands to more open areas as this provides them with better breeding habitat, which explains their higher abundances in the east than the west (Hatchwell, Chamberlain and Perrins, 1996; von dem Bussche et al., 2008). The distribution patterns relating to soil type may be due to indirect effects from relationships between soil and landscape features, or there may be more direct effects. For example, food availability may be a factor, since earthworm density is related to soil type (Rutgers et al., 2016). Other studies looking at mallard distribution have consistently found a strong positive relationship between abundance and the presence of water bodies and wetlands (Milsom et al., 2000; Newbold and Eadie, 2004; Barker, Cumming and Darveau, 2014; Herbert et al., 2018). While we found a positive relationship with all water and wetland types, this was not a particularly influential factor in our model. We hypothesise that this is because the Netherlands is a very wet country with a huge number of very small water bodies, particularly in the west, meaning that there are few areas which are far from water (Het Waterschapshuis, 2017). The high abundances of mallards in the west also fits with the results of (Barker, Cumming and Darveau, 2014), who found that mallards prefer areas with high water body density and open grassland. The preference of house sparrows for urban areas has been well documented. They nest in buildings and urban green spaces provide good food availability (Robinson, Siriwardena and Crick, 2005; Murgui, 2009; Šálek, Riegert and Grill, 2015; Bernat-Ponce, Gil-Delgado and Guijarro, 2018). (Ramírez-Cruz and Ortega-Álvarez, (2021) also note that house sparrows likely avoid natural areas with high vegetation due to limited food resources, difficulty spotting predators and competition with other species. Their preference for the eastern parts of the country over the west could be attributed to the sandier soil in the east, since house sparrows like to take sand baths (Hauser, 1957; Pandian, 2023).

Blackbird abundance is expected to stay roughly the same in the future, while the abundance of mallards and house sparrows is expected to increase. Looking at the partial dependence plots (see supplementary materials), the largest differences between the three species are in the vegetative cover, particularly at the smaller buffer sizes. It is possible that blackbirds are responding differently to future changes in grass and tree cover than mallards and house sparrows and this is why we see the different changes in abundance. Since the vegetative cover variables were found to be particularly important, and all three species respond similarly to changes in climate and land use, this seems to be the most plausible explanation. There are only small differences in bird abundance between the four scenarios. This is possibly because we only look thirty years into the future, which means they have not had so much chance to diverge. Also, all the scenarios have similar trends: warmer temperatures, less agriculture and increasing urbanisation, but to different extents. It therefore makes sense that we see similar patterns in all the scenarios. This is helpful for controlling disease risk, since we have a good idea how bird populations will change, regardless of the scenario, and can plan accordingly.

In terms of what this means for disease risk, there are many factors to consider. All three species studied had a positive relationship between abundance and urban areas and the Netherlands is expected to become more urban in future. Research on avian disease in urban areas has produced mixed results. Several studies have shown reduced pathogen prevalence in urban blackbirds when compared with their rural counterparts (Geue and Partecke, 2008; Evans et al., 2009). On the other hand, other studies have found that while avian disease risk is reduced in cities for some diseases, it increases for others (Martin and Boruta, 2014), for example, for flaviviruses such as WNV, which can be transmitted by all three species considered here. Figuerola et al., (2024) propose that urbanisation provides opportunities for different rather than reduced pathogen transmission, as well as facilitating new interactions between vectors, hosts and parasites. The abundance of mallards and house sparrows is predicted to increase, providing additional potential disease hosts. It therefore seems prudent to regularly monitor bird populations in urban areas for diseases, particularly for flaviviruses and other pathogens which are known to benefit from an urban environment.

Our case study on USUV in blackbirds suggests that higher bird abundance is linked to higher disease risk. Indeed, avian abundance has been found to be positively related to disease prevalence for several pathogens, though there are exceptions and it should not be assumed that this will always be the case (States, Hochachka and Dhondt, 2009; Zhang et al., 2014; Santiago-Alarcon et al., 2016; Ellis et al., 2017). The increase in mallard abundance could have a particularly large effect, since they travel relatively large distances both nationally due to breeding dispersion (up to 19 km) and internationally through migration, leading to a greater risk of diseases spreading to new areas (van Turnhout, 2011; Bartel et al., 2018). The highest increases in abundance were in areas which currently have low abundance, suggesting an ‘evening-out’ effect. This could potentially make it harder to predict areas which are at particularly high risk of disease outbreaks and necessitate more widespread surveillance than would otherwise be the case. There are also ongoing changes in bird migration patterns due to climate change, which we have not considered in this study. Both migration times and destinations are changing, potentially exposing birds to pathogens they would not otherwise have been exposed to (Pautasso, 2012; Hall, Brown and Altizer, 2016). These changes create additional challenges for surveillance programmes, but also make such programmes all the more important. Increased host abundance, combined with higher uncertainty on potential outbreak locations and greater risk of new disease introductions, makes widespread avian disease surveillance vital if new outbreaks are to be detected in a timely manner.

## Conclusions

Blackbirds, mallards and house sparrows are all likely to be especially relevant for disease transmission in Northwestern Europe and are capable of transmitting a wide range of pathogens. Their abundances will likely increase in future, especially for mallards and house sparrows. Unfortunately, it is not possible to extrapolate these results to other bird species, since they will respond differently to future climate and land-cover changes. However, it is interesting to note that while many bird species are expected to decline in future (Huntley et al., 2008; Soultan et al., 2022; Rigal et al., 2023), these three species which are potentially key for disease transmission will thrive. Quantifying the consequences of these abundance changes is complicated as there are many factors to consider; however, it is likely that increased pathogen reservoirs will increase disease risk and that changes in distribution may affect local outbreak risk. Future research could use the detailed abundance maps created in this study to model future disease risk. This could then inform disease surveillance work and contribute to outbreak preparedness.

## Data Availability

All model inputs (with the exception of the bird data), model outputs, code and supplementary information can be accessed via the Dryad repository: https://doi.org/10.5061/dryad.r2280gbmc. The bird data are not publicly available but can be requested from the Dutch Centre for Field Ornithology (Sovon: sovon.nl) or Vigie-Nature (https://www.vigienature.fr/fr/suivi-temporel-des-oiseaux-communs-stoc). A summary of the bird data is available via the above Dryad link.
